# Plastic frontal pole cortex structure related to individual persistence for goal achievement

**DOI:** 10.1038/s42003-020-0930-4

**Published:** 2020-04-28

**Authors:** Chihiro Hosoda, Satoshi Tsujimoto, Masaru Tatekawa, Manabu Honda, Rieko Osu, Takashi Hanakawa

**Affiliations:** 1 PRESTO, Japan Science and Technology Agency, Kawaguchi, Saitama, 332-0012 Japan; 2 Department of Life Science Graduate school of Arts and Sciences, The University of Tokyo, Tokyo, 153-8902 Japan; 3 Department of Motor Control and Rehabilitation, ATR Computational Neuroscience Laboratories, Kyoto, 619-0288 Japan; 4 Department of Information Medicine, National Institute of Neuroscience, National Center of Neurology and Psychiatry, Tokyo, 187-8502 Japan; 5 Department of Advanced Neuroimaging, Integrative Brain Imaging Center, National Center of Neurology and Psychiatry, Tokyo, 187-8551 Japan; 6 Strategic Innovation Research Center, Teikyo University, Tokyo, 173-8605 Japan; 7 The Nielsen Company Singapore Pte. Ltd., Singapore, 228233 Singapore; 8 Shibaura Institute of Technology Graduate School of Engineering and Science, Tokyo, 108-8548 Japan; 9 Faculty of Human Sciences, Waseda University, Saitama, 359-1192 Japan; 10 Department of Integrated Neuroanatomy and Neuroimaging, Kyoto University Graduate School of Medicine, Kyoto, 606-8303 Japan

**Keywords:** Cognitive control, Motivation

## Abstract

Persistent goal-directed behaviours result in achievements in many fields. However, the underlying neural mechanisms of persistence and the methods that enhance the neuroplasticity underlying persistence, remain unclear. We here demonstrate that the structural properties of the frontal pole cortex (FPC) before tasks contain information that can classify Achievers and Non-achievers (goal-directed persistence) participating in three tasks that differ in time scale (hours to months) and task domains (cognitive, language, and motor learning). We also found that most Achievers exhibit experience-dependent neuroplastic changes in the FPC after completing language and motor learning tasks. Moreover, we confirmed that a coaching strategy that used subgoals modified goal-directed persistence and increased the likelihood of becoming an Achiever. Notably, we discovered that neuroplastic changes in the FPC were facilitated by the subgoal strategy, suggesting that goal-striving, using effective coaching, optimizes the FPC for goal persistence.

## Abstract (web summary)

Hosoda et al. study the neurobiological underpinnings of goal pursuit and persistence. They use MRI data and identify areas in the frontal pole cortex that could predict performance on various tasks. They also show that coaching results in neuroplastic remodeling that increases the likelihood of goal persistence.

## Introduction

“Mikka Bozu” (a three-day monk) is a widely used Japanese idiom that refers to a person who easily gives up. In Japan, which at one time was a devoutly Buddhist society, the social status of a high-ranking monk was considerable, prompting many to attempt to join the monastic ranks. However, despite an initially high degree of idealism and enthusiasm, many would-be monks were not able to tolerate monastic training and the secluded lifestyle; therefore, they would abandon their training after only a short period. People who lack the necessary persistence and miss opportunities—such as a three-day monk—can be found almost anywhere in the world. The ability to pursue goals effectively is critical for attaining happiness and well-being in life^1^. One way to live a happy and satisfying life is to pursue things that an individual may consider to be of high value, such as the acquisition of fame or fortune, academic achievement, success in athletic competition, diet, and exercise. These goals are not readily or immediately attainable but instead are attained because of persistent efforts.

Unfortunately, many people give up in the middle of this pursuit. Studies reveal that the failure rate in the achievement of long-term, goal-directed behaviors, such as dieting^2^, smoking cessation^3^, rehabilitation^4^, and acquisition of a foreign-language^5^, is ~50%. This occurs despite the fact that people are aware of the importance of persistence for success. Even though it is evident that persistent work is necessary for fulfilling achievements, just knowing is not sufficient since we still face motivational and external constraints^6^.

Persistence is a critical high-order ability that is independent of task or context^7–9^. In line with its high-order nature, many factors have been identified as being contributory to the achievement of a task or goal. These factors include self-control^8,10–13^, motivation^14^, conscientiousness/grit^15^, and self-estimation^16^. It is known that coaching methods and strategies, such as setting subgoals, can increase net achievement in learning^17,18^. Thus, many researchers question how an individual’s persistence can be trained when it is under the influence of such complex factors. It is no exaggeration to say that strengthening one’s persistence is a universal desire. Therefore, it would be beneficial to clarify the neural basis that underlies persistence/giving-up and to develop a method that enhances persistence by inducing neuroplasticity.

Multiple studies have demonstrated that the frontal pole cortex (FPC) underlies goal-directed persistence ^19–22^. The FPC in humans is considerably larger than that in other animals^23,24^. In the hierarchical organization of the frontal cortex, the FPC, especially the lateral area, is suspected to be important for schematic control of behavior (i.e., behavioral control based on an internal state)^25^. Although there is no direct evidence for its involvement in persistence, the FPC has been implicated in several functions that are relevant to goal achievement, including the prospection of future events^26^ and outcomes^27–30^, motivating cognitive and physical effort^31^, and metacognitive control^32^. This area also plays a role in managing a sequence of subgoals at both the sub- and super-ordinate levels^33,34^ and may underlie the modification of persistence for learning in behavioral observations^17,18^.

The present study aimed to address these hypotheses from a brain-structural perspective. We first performed three experiments that differed in time scales (hours to months) and task domain (cognitive, language, and motor learning) to test whether specific brain areas, particularly the FPC, predicted persistence across different types of behaviors at an individual level. Next, we used a measure of structural neuroplasticity that underlies several types of learning^5,35–41^ to investigate whether coaching strategies (i.e., subgoal setting) affected the neural basis of persistence and thereby modulated goal-directed persistence.

## Results

### Left FPC structure predicts persistence in a planning task

To assess persistence, previous studies have employed long-term behaviours that require months to years to complete^2–4,42,43^. The commonality and specificity of persistence from an hour to months were of interest here. Therefore, in the present study, we first tested persistence using the Tower of Hanoi (ToH) task, which can be completed in approximately an hour. ToH is a cognitive task that is widely used to test the frontal lobe functions, including problem-solving, planning, and executive functions^44^. We tested whether participants could complete the task (Achiever) or not (Non-achievers) without time limitations.

**Fig. 1:**
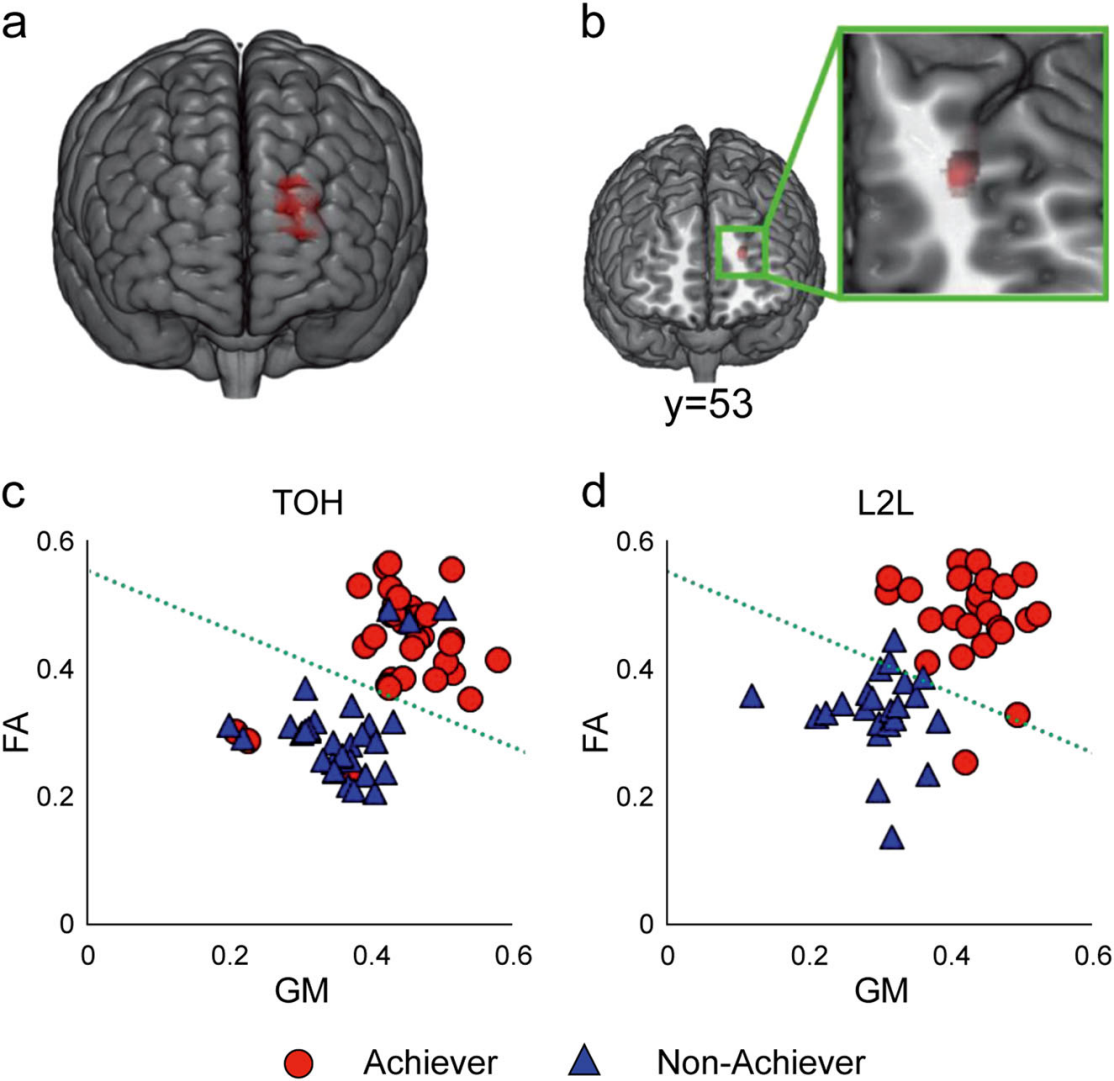
Developed left frontal pole cortex with organized fibre connectivity in Achievers. **a** The GM volume was greater in the left FPC of Achievers than that in the left FPC of Non-achievers during the ToH task (*p* < 0.05, FWE-corrected). **b** More organized fibre connectivity was observed beneath the left FPC in the Achievers than that in the Non-achievers (*p* < 0.05, FWE-corrected). **c** A linear classifier was trained based on GM and FA values before the ToH task. The green broken line indicates the dividing line between the Achievers and the Non-achievers. The classification accuracy was 90.9% for the ToH task (using leave-one-out cross-validation). **d** The ToH-based persistency detector was also able to classify Achievers and Non-achievers in an independent cohort that was enrolled in an L2L program with a classification accuracy of 80.7%.

We enrolled 65 university students (29 women, 22.5 ± 5.3 years, range 20–28) to perform the 7-disc version of the ToH task. Before the task, the participants were instructed to predict the amount of time they would require to complete the task (predicted time). As expected, not all participants completed the ToH. Among the participants, 34 were Achievers and 31 were Non-achievers. On average, Achievers completed the ToH in 29.4 min (SD = 10.5 min), while Non-achievers declined to complete the task after 14.3 min (SD = 7.1 min). Reasons for declining were “harder than expected” (n = 28) and “tired” (*n* = 3). We analysed the participants’ brain structure using T1-weighted magnetic resonance imaging (MRI) and diffusion-weighted MRI^45^ before the task. Differences in brain structure across the whole brain were compared between the Achievers and the Non-achievers. More precisely, we performed a general linear model (GLM) analysis and incorporated not only the groups but also sex, age, motivation score, and prediction time as explanatory variables in order to remove the effects of these factors from the independent imaging variables. Compared to Non-achievers, the grey matter (GM) volume in the left FPC in Achievers was significantly greater (*p* < 0.05, family-wise error [FWE]-corrected) (Fig. 1a, Supplementary Table 1). There was also significantly more organized fibre connectivity beneath the left FPC in Achievers than the Non-achievers (*p* < 0.05, FWE-corrected) (Fig. 1b). Since the MRIs were obtained before participants completed the ToH task, these findings suggest that individual differences in the left FPC structure may have a predictive value for judging whether a participant would complete the ToH task or not.

We constructed a classifier based on the two anatomical parameters to test the possibility of predicting goal-directed persistence using the FPC structure. We set up a 5 mm spherical volume-of-interest (VOI) at the peak voxel in the left FPC for both the GM and the fractional anisotropy (FA) analyses. Next, we extracted both the GM and the FA values from the left FPC as regional features that exhibited group-wise differences (see Methods for details). The Fisher’s linear discriminant classifier was trained based on that data, generating a binary value that indicated whether the structural pattern of the FPC belonged to Achievers or Non-achievers. Based on the ToH task, this persistency detector identified Achievers and Non-achievers with an accuracy of 90.9% (leave-one-out cross-validation, *p* = 0.001 by binomial test) (Fig. 1c, Supplementary Table 2). This result was confirmed using a 2-fold cross-validation and bootstrap method (accuracy = 88.2%). We also tested a classifier based on the right FPC structure (see Methods for details), which had an accuracy of 63.0% and did not differ from the chance level (*p* = 0.98 by binomial test). This further analysis demonstrated the specificity of the left FPC in predicting persistence.

### Generalization of persistency detector to other tasks

We tested the performance of the left FPC structure-based persistency detector that was trained on the ToH task retrospectively in a validation cohort from our previous second language learning (L2L) study^5^. Importantly, since this task lasted for several months, we were able to assess whether the persistence detector was applicable to a task that required long-term persistence. In the L2L experiment, 23 of 45 participants dropped out in the middle of the course; however, we had not previously analysed the data from the Non-achievers. Compared with the Non-achievers, the Achievers had a significantly greater GM and more organized fiber connectivity beneath the left FPC (*p* < 0.05, FWE-corrected) (Supplementary Fig. 1). We applied the ToH-derived persistency detector retrospectively to discriminate Achievers from Non-achievers in this previous task using their initial structural MRIs and found that it had a classification accuracy of 91.1% (*p* < 0.001 by binomial test), sensitivity of 91.2%, and specificity of 90.3% (Supplementary Table 3). This accuracy was higher than expected as the areas involved in each task (L2L^5^ or ToH^44^) and the task durations were different. Nevertheless, these analyses demonstrate that the neural basis of persistence mostly overlapped. Thus, despite being retrospective, this finding further supported the suggestion that the left FPC structure predicts persistence. To complete our argument, we applied the right-FPC-based persistency detector to L2L but only observed chance-level of discrimination rates (43.7%).

Next, we prospectively tested the performance of the persistency detector in a second validation cohort in which the learning content was different from that in the ToH and L2L tasks. We used motor sequence learning (MoL) that involved frontal lobe functions that were distinct from the ToH and L2L tasks and a longer learning duration than that of the ToH task. The MoL experiment was designed to test whether the ToH-derived left FPC classifier was able to predict prospectively persistence in a wide range of learning tasks that were independent of the learning content.

Forty university students (20 females) with a mean age of 22.5 years (SD = 5.3; range = 20–28) were enrolled in a 5-week computer-based motor learning task that involved sequential finger tapping. Each day, the participants were instructed to learn a new tapping sequence that consisted of 10 movements by trial-and-error. The goal of the day (the word “clear”) was displayed only after the participants successfully executed 10 successive correct new tapping sequences at 2 Hz after performing all previously learned sequences without any mistakes (long-term goal [LG]).

**Fig. 2:**
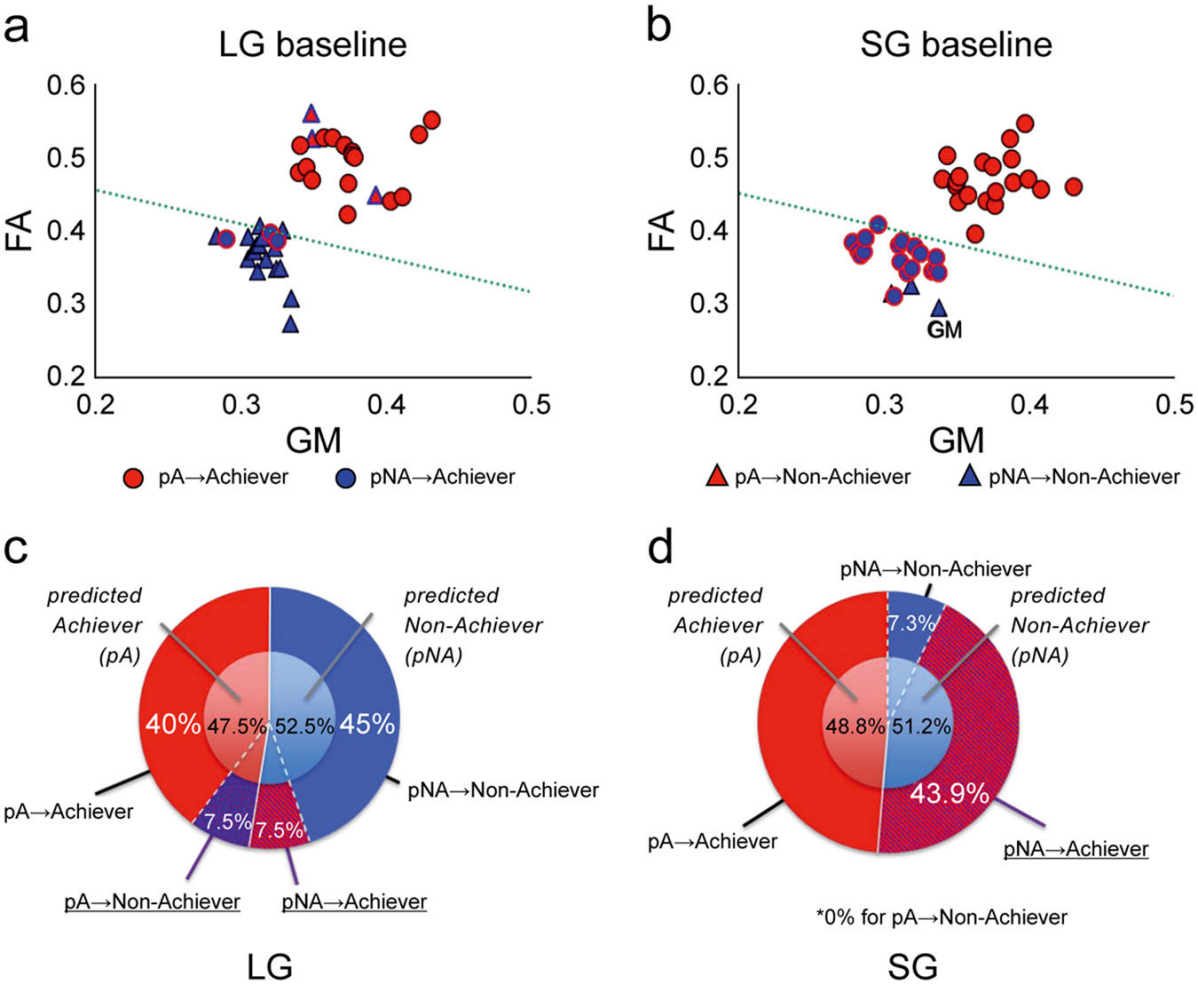
Persistency detector prospectively predicted goal-directed persistence in MoL without subgoals (LG) but not in MoL with subgoals (SG). a The “persistency detector” prospectively predicted Achievers and Non-achievers in the LG group with an accuracy of 85 % (p < 0.01 by binomial test). **b** The “persistency detector” failed to prospectively predict Achievers and Non-achievers in the SG group (accuracy = 56 %; *p* = 0.17). **c** The inner circle represents predicted persistence, while the outer circle indicates actual persistence in the LG (**c**) and SG (**d**) groups. The “persistence predictor” predicted a similar proportion (~50%) of predicted Achievers (pA) and Non-achievers (pNA) across the groups before MoL. The actual number of Achievers and Non-achievers after MoL differed between the LG and SG groups (chi-squared test, *p* < 0.001). As predicted, 19 participants were Achievers and 21 were Non-achievers in the LG group. The persistence predictor was incorrect in only six participants (14%). **d** Additionally, 38 participants (92.7%) were Achievers and only three (7.3%) were Non-achievers in the SG group. Namely, 86% (18/21) of pNA converted to Achievers.

We applied the ToH-derived persistency detector to the structural MRIs that were obtained before the MoL task to predict prospectively Achievers and Non-achievers. Before the intervention, the detector predicted that 19 participants would complete the program (predicted Achievers, pA) and that 21 subjects would not complete the program (predicted Non-achievers, pNA). The actual results indicated that 16 participants completed the MoL program while 24 did not. The persistence detector prospectively predicted Achievers and Non-achievers with an accuracy of 85% (*p* < 0.001 by binomial test), sensitivity of 84.2%, and specificity of 85.7% (Fig. 2a, Supplementary Table 3). We also applied the right-FPC-based persistency detector to MoL but only observed chance-level of discrimination rates (46.5%).

These findings suggested that the performance of the persistency detector could be generalized to a wide range of tasks. All of the Non-achievers quit the learning task within 3 days due to the following reasons: “harder than expected” (*n* = 19), “too busy” (*n* = 2), “being bored” (*n* = 2), or “eyes hurting” (*n* = 1). Although the persistency detector initially classified these three Non-achievers as Achievers, one of the participants failed to become an Achiever because of a physical problem (eyes hurting) and the other two because of a mismatch between their ability preference and the task difficulty (harder than expected). No differences were observed in self-rated motivation, sex, or personal traits between the Achievers and the Non-achievers (Supplementary Table 4).

Finally, the persistency detector, developed from the ToH experiment data, may have included factors specific to the task. To remove these confounding factors and to avoid the problem of over fitting, Fisher’s Linear Discriminant was performed using data from the ToH, L2L, and MoL-LG tasks as a pooled dataset. In total, 144 samples were randomly divided into groups of 101 (70%) and 43 (30%): 70% of the samples were treated as a training dataset, and the 30% of the samples were treated as a test dataset. The detector performance was evaluated using the bootstrap method (1000 times). This new detector showed an accuracy of 89.61% (Supplementary Table 5).

### Short-term subgoals enhance persistence

Previous studies have shown that setting subgoals enhances net achievement in learning^17^. Here, we tested if setting subgoals would also enhance goal-directed persistence using the MoL task.

We enrolled 41 university students (21 females) with a mean age of 22.8 years (SD = 5.1, range = 20–26) to participate in a similar MoL program but with different goal settings (i.e., coaching strategy). In this group, an indication of the achievement of a subgoal (“clear”) was displayed after two or three successive correct responses during the trial-and-error phase (short-term subgoal [SG]). Before the training, we assessed the left FPC structure of the students using the ToH-derived persistency detector. According to the detector, 21 of the 41 participants (SG group) were predicted as Non-achievers (pNA) and 20 were predicted as Achievers (pA). There was no difference in the distribution of pA and pNA between the LG and SG groups (chi-squared test, *p* = 0.908, *χ*^2^ = 0.013). Moreover, there were no significant differences in sex, age, performance IQ, typing test score, or personal traits between the SG and LG groups. Further, there were no differences in these scores between the Achievers and Non-achievers (Supplementary Data 1).

Although three predicted Non-achievers quit the program within 3 days due to it being “harder than expected,” the other 38 participants in the SG group completed the MoL program regardless of the predicted outcome. Thus, all pA were true Achievers, but only three (7.3%) out of the 21 pNA were true Non-achievers (Fig. 2d). In other words, 86% of the pNA converted to Achievers (converted pNA) when they performed the MoL task with short-term subgoals. In contrast, only six participants (5%) in the LG group converted from their predicted category (Fig. 2c). Accordingly, the prediction accuracy was significantly lower (chi-squared test, *p* = 0.001, *χ*^2^ = 11.952) in the SG group (56%) than that in the LG group (85%) (Fig. 2a, b).

No pauses were inserted after subgoals (presentation of “clear”) in the SG group; thus, their MoL progress was similar to that of the LG group. Indeed, there was no difference in the total training time between the Achievers in the LG group (69.4 ± 13.9 h) and those in the SG group (65.3 ± 14.1 h) (*p* = 0.43). On the one hand, the behavioral results supported our hypothesis that the coaching strategy with a subgoal setting would enhance goal-directed persistence. On the other hand, we failed to predict pNA in the SG version of the MoL task. The only difference was that several subgoals were provided to the SG group.

### Enhanced neuroplastic changes in left FPC in subgoals learning

**Fig. 3:**
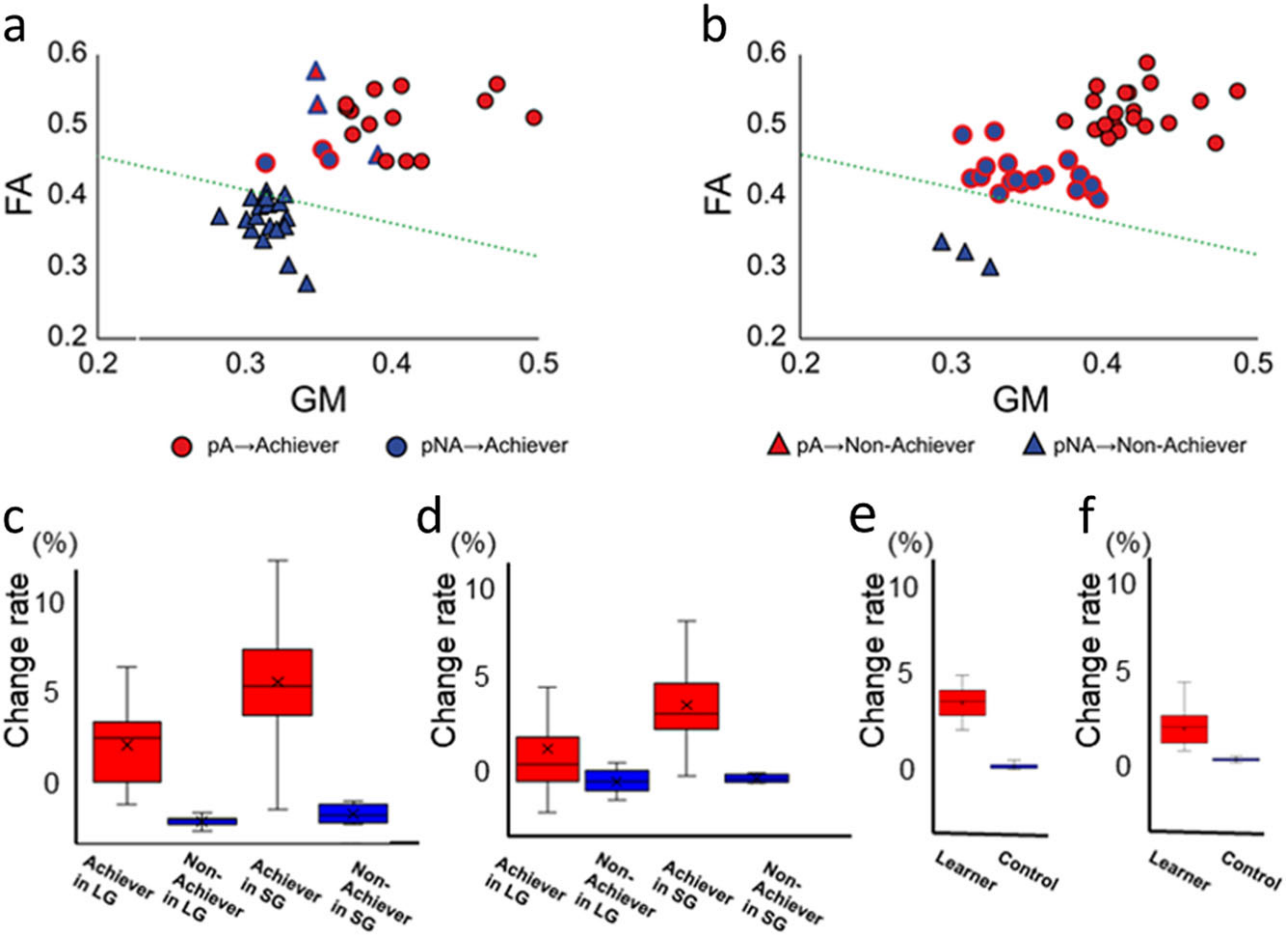
Persistency detector applied to MRI after MoL and neuroplastic changes in the left FPC induced by different coaching strategies. The persistency detector applied to the left FPC structure after MoL classified Achievers and Non-achievers in both the LG (**a**) and SG (**b**) groups. Most pNA → Achiever converters (blue circles with red circumference) after MoL were classified as Achievers in both the SG and LG groups. **c** MoL-induced increases in GM (% changes). A two-way ANOVA revealed STRATEGY × PERSISTENCE interactions (*F(*_3,77)_ = 13.03, *p* < 0.001), indicating that GM was increased in the SG group as compared with that in the LG group. **d** Similarly, there were significant STRATEGY × PERSISTENCE interactions (*F(*_3,77)_ = 16.64 *p* = 0.001) in MoL-induced increases in FA (% changes); therefore, FA was increased in the SG group as compared with that in the LG group. **e** L2L-induced increases in GM (% changes) in the Achievers and non-trained controls. The two-sample *t*-test indicated that there were significant changes in the GM of the FPC before and after participants completed the L2L program (Achievers) as compared with those in the controls (*p* = 0.001). **f** Similarly, a two-sample t-test indicated that there were significant post-minus-pre changes in the FA beneath the FPC in L2L learners (Achievers) as compared with those in the controls (*p* = 0.002).

To gain insights into the neural mechanisms that underpin the conversion from pNA to Achievers, we analysed the structural MRIs of participants in the SG and LG groups that were obtained after MoL training. Contrary to the *a priori* classification, the FPC structures from all converted pNA were classified correctly as belonging to Achievers after SG-MoL (Fig. 3a, b, Supplementary Tables 3 and 4). This result suggested that the original Non-achievers-type FPC structure may have changed to the Achiever-type structure during the MoL task with SG in the converted pNA-Achievers. Therefore, we hypothesized that the achievement of subgoals may have modulated the structural properties of the FPC and thereby converted the classification category from the Non-achiever type to the Achiever-type. Although structural neuroplasticity following goal achievement remains to be elucidated, it is reasonable to assume that the FPC undergoes neuroplastic changes that are associated with a training program that leads to improved persistence^46,47^.

Indeed, several types of learning induce neuroplastic reorganization of structural properties in task-relevant regions of the brain^5,37,38,48,49^. Therefore, we directly investigated the differences in the neuroplastic changes in the FPC structure induced by SG and LG. We retrieved GM and FA values before and after the MoL task from VOIs in the left FPC to assess potentially learning-induced changes in GM and FA. We performed a two-way ANOVA using STRATEGY (LG and SG) and PERSISTENCE (Achievers and Non-achievers defined after MoL) as between-subject factors. There was a significant main effect of PERSISTENCE (*F* (3,77) = 85.59, *p* = 0.001) and a significant STRATEGY × PERSISTENCE interaction effect (*F* (3,77) = 13.03, *p* = 0.001) on changes in GM volume. For the FA changes ([FApost – FApre]/FApre), we also observed significant STRATEGY × PERSISTENCE interactions (*F* (3,77) = 18.37, *p* = 0.001) and main effects of PERSISTENCE (*F* (3,77) = 22.03, *p* < 0.001). These results indicated that neuroplastic changes in the left FPC differed between the Achievers and the Non-achievers (main effects) partially depended on the difference in the coaching strategy in the SG and LG groups (interactions). These results supported the hypothesis that achieving subgoals facilitated neuroplastic changes in the left FPC that are associated with goal-directed persistence. Furthermore, we analysed changes in the structural properties (GM and FA) of the FPC before and after the L2L task to test the generalization of learning-induced neuroplasticity in the FPC. Results from a two-sample t-test (Fig. 3e and f) revealed that GM (*p* = 0.001) and FA (*p* = 0.002) were increased in the L2L Achievers compared to those in the controls who did not complete L2L (no post-learning MRI data from Non-achievers). This analysis further indicated that neuroplastic changes in the FPC were associated with persistent engagement towards a goal. It should be noted that potential learning-induced neuroplastic changes in the FPC were especially pronounced with the addition of subgoals. That is, even though neuroplastic changes occurred in both the LG and SG groups, the magnitude of changes in the SG group was greater than that in the LG group. This reflected the fact that the converted pNA-Achievers were mostly from the SG group.

**Fig. 4:**
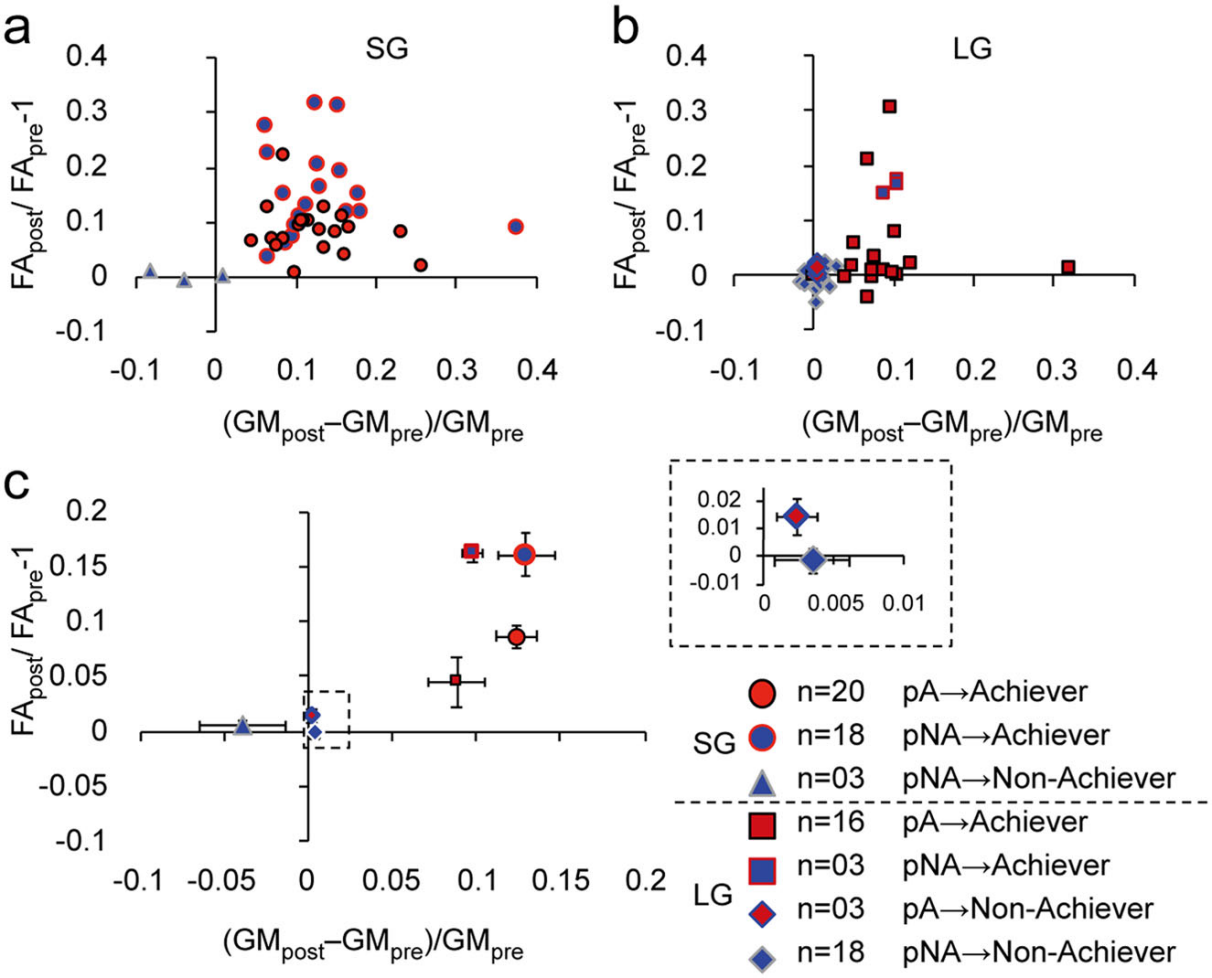
GM and FA changes after motor sequence learning in the left FPC. Changes in GM and FA before and after MoL in each individual in the LG (**a**) and SG (**b**) groups. No changes were observed in the Non-achievers in either group regardless of prediction (pA or pNA) before the experiment. **c** Group average of GM and FA changes after learning. We only focused on changes in the Achievers (see text). A two-way repeated measures ANOVA revealed a main effect of STRATEGY (SG, circle vs. LG, square) on GM. The SG group demonstrated a greater increase in GM. An ANOVA revealed main effects of STRATEGY and PERSISTENCE (pA vs. pNA) on FA. The SG group exhibited a greater increase in FA. Additionally, FA was increased in the pNA as compared with that in the pA.

To explore this phenomenon from another perspective, we tested whether the pA and the pNA experienced different neuroplastic changes depending on the coaching strategy (SG vs. LG). We focused on changes in GM and FA in the Achievers since the statistical results of Achievers (Fig. 3) revealed both STRATEGY × PERSISTENCE interactions and the main effect of PERSISTENCE. Overall, the FA increased in converted Achievers (pNA → A) compared to that in original Achievers (pA→A) (Fig. 4a, b for individual data and 4c for group-averaged data). However, we observed different effects of PREDICTION (pA and pNA) and STRATEGY (SG and LG) between GM and FA. For GM changes, a two-way ANOVA that used PREDICTION (pA and pNA) and STRATEGY (LG and SG) as between-subject factors revealed main effects of STRATEGY (F(1,53) = 5.215, *p* = 0.026), but there was no main effect of PREDICTION (F(1,53) = 0.834, *p* = 0.37) or interactions (*F*(1,53) = 0.488, *p* = 0.488). For FA, we observed main effects of STRATEGY (F(1,53) = 5.8, p = 0.019) and PREDICTION (*F*(1,53) = 6.41, *p* = 0.014) but there were no significant interactions (*F*(1,53) = 0.47, *p* = 0.829). In summary, learning with subgoals induced greater GM and FA changes than those induced by learning without subgoals, regardless of prediction. The conversion from pNA → Achiever demonstrated a closer relationship with FA changes than GM changes regardless of the implementation of subgoals. In other words, neuroplastic changes that were involved in the conversion from pNA → Achiever were more likely linked to the reorganization of white matter than to the reorganization of GM.

## Discussion

We demonstrated that the structural properties of the FPC contain information that predicts goal-directed persistence across multiple tasks. The “persistency detector” generated from the structural properties of the left FPC accurately classified Achievers and Non-achievers who were enrolled in a ToH task. Moreover, this “persistency detector” was generalizable to validation cohorts that were enrolled in an L2L program and the LG version of a MoL program. Moreover, we demonstrated that coaching strategies modified goal-directed persistence. Setting subgoals increased the number of Achievers and reduced the number of Non-achievers, and many pNA achieved the goal in the SG version of MoL. Although this may indicate that the persistency detector failed to predict Achievers and Non-achievers in this cohort, we observed that most of the converters belonged to the SG group. This finding suggests that the structural properties of the FPC that are related to goal-directed persistence are at least partly experience-dependent. We also confirmed experience-dependent GM and FA neuroplastic changes in the FPC after completing the MoL and L2L programs. Neuroplastic changes in the FPC were most pronounced in the participants in the SG group of the MoL cohort but were also observed in all types of Achievers. The pNA → Achiever conversion was especially associated with FA changes, which were not detected in the Non-achievers or controls.

A growing body of evidence has indicated that the FPC is consistently active and is necessary for monitoring and processing the value of alternative options and actions^27,50^. In line with these functions, a recent study reported that the upregulation of FPC excitability with anodal transcranial direct current stimulation enhanced the willingness to exert mental and physical efforts to achieve greater rewards^51^. This implies that high-cost, high-benefit options that tend to be rejected become more attractive with increased FPC excitability^51^. Achieving a goal through persistent effort is typically accomplished by removing low-cost, low-benefit options from the selection of available alternatives^52^. Thus, the FPC may underlie goal-directed persistence by regulating the choice space through precommitment.

This hypothesis is in accordance with the well-known hierarchical organization of the frontal cortex, which was recently refined by Badre and Nee^25^. While the FPC has long been thought to sit at the apex of the hierarchy, Badre and Nee alternatively proposed that the anterior part of the dorsolateral prefrontal cortex (DLPFC), rather than the FPC, is the apex of the hierarchy^25^. Importantly, in this newly proposed model, the FPC specifically processes internal schema-based information that is possibly transmitted from the ventromedial PFC and its associated network and sends output to the DLPFC where sensory information from posterior areas converges with the schematic information from the FPC. Through this system, the FPC may communicate with the regions that are involved in the control of reward-related impulses, such as the DLPFC, at the time of precommitment decisions^53^, thereby removing tempting but ultimately less beneficial alternatives from the current choice space and promoting patience for greater achievement in the future^54,55^. This hypothetical role of the FPC in goal-directed persistence concurs with our findings that the utility of the ‘persistency detector’ was generalizable across diverse tasks with different time scales that ranged from hours to months and across different domains, such as cognitive, language, and motor learning.

This hypothesis is also consistent with the classic findings that damage to the anterior PFC, including the FPC, results in impairments in planning^26,56,57^. Complex planning derives in part from the faculty to plan an uncertain but potentially controllable future^58^, and deficits in control of impulsive choices would result in suboptimal planning. In this context, a complex plan is merely a plan until it is fully executed and tested for its feasibility. Without execution, the capacity of complex planning receives no feedback from reality and cannot be properly trained. The more complex the planning, the more persistence is required for achieving its realization. This concept is consistent with the fact that expert skill acquisition in any field (except sports) relies on persistent training rather than genetic factors^59–61^. Therefore, we argue that the backbone of goal-directed persistence is the faculty of complex planning, to which the FPC contributes via regulation of the choice space through precommitment. This faculty is properly trained with positive feedback through the experience of achievement.

In the hierarchical model of approach motivation, system, strategic, and tactical levels of motivation were considered to be instantiated along an anterior-to-posterior gradient of the superior lateral prefrontal cortex (including Brodmann Area 10)^62^. Further, studies have also suggested that the orbitofrontal cortex (OFC) and anterior cingulate cortex (ACC) provide information about stimulus and action value, respectively, to these areas. Lastly, the frontopolar PFC (including Brodmann Area 10), was thought to be involved in branching control (i.e., maintaining a final goal in memory while another task that required persistence was carried out)^62^. Thus, we propose that these frontal networks are involved in goal-oriented behavior and that the FPC may play a role as a hub to maintain goal-directed persistence via regulation of the choice space through precommitment.

An important finding in this study was that setting subgoals enhanced both behavioral goal achievement and neural plasticity in the FPC. Past studies also proposed the term ‘cognitive branching’ to describe the involvement of the FPC in situations that require the maintenance of primary task goals (supraordinate) while simultaneously allocating attention to subgoals (subordinate)^22,32,63–66^. However, it was unclear whether cognitive branching (subgoals) would be required for primary goal achievement as the task design utilized by past studies was novel and complex. In the current study, we observed that subgoals enhanced primary goal achievement and FPC plastic changes. However, one limitation was that we could not test whether the pNA→Achiever converters were able to achieve goals on other occasions. The FPC may be primarily engaged under conditions where the cost-benefit computation of the ultimate goal choice can be supported by integration with information regarding subgoals. Hence, the primary factor that influences the FPC does not appear to be the subgoal processing itself but rather the requirement to integrate these two sources of information. The FPC also appears to be related to the processing of reward-related information^67,68^. The FPC may monitor the rewarding value of an ongoing task and integrate information to update the complex planning of goal-directed behaviors.

We observed that while the FPC structure before learning prospectively predicted the participants who would become Achievers, persistent daily training induced neuroplastic changes in the FPC structure. This experience-dependent neuroplasticity coincides with previous findings that suggest that neuroplastic changes were accompanied by improvement in cognitive ability^5,35–41^. Importantly, a recent study revealed that the lateral FPC is specifically involved in the metacognitive control of decision adjustment in situations where no explicit feedback is available^31^. The FPC of predicted achievers (pA) may have developed this function through past experiences that allowed them to remain Achievers even without subgoal instruction. In contrast, the pNA may not have sufficiently developed the FPC to support the metacognitive control that was required for the present learning task; hence, they may have benefitted from coaching with systematic explicit feedback. This may be the reason why neuroplastic changes in the FPC occurred primarily in the pNA→Achiever participants. Thus, the present findings suggest that postnatal training induces neuroplastic changes in the FPC structure and thereby improves its function in goal-directed persistence. Additionally, the setting of appropriate subgoals and feedback are beneficial for these processes.

An increase in the GM and FA values is correlated with performance improvement from training^38,41^. Although the underlying neurobiological mechanisms have yet to be revealed, the proposed factors for changes in FA include the proportion of crossing fibres^69–71^, axonal permeability^62^, and cell density or axonal/dendritic arborisation^41,71^, while those for GM changes include neurogenesis, gliogenesis, synaptogenesis, and vascular changes. However, the exact biological substrates underlying the changes in GM and FA are still under investigation, and the mechanisms underlying the increase in FA values, especially in the converters, require future investigation.

Notably, the neuroanatomical basis of persistence was lateralized to the left FPC. In all three experiments, the Achievers were likely engaged to achieve the desired state (approach motivation), not to avoid the undesirable state (avoidance motivation) since there was no punishment in the task. Therefore, the development of left PFC in the Achievers was consistent with reports of the goal theory of neurobiology^72–76^, which shows that the self-regulatory process of hierarchical control can be mapped onto the prefrontal cortex (especially in the left hemisphere) in association with approach motivation^74^.

The present experimental paradigm examined goal approach behaviour rather than goal avoidance behaviour. The approach goal pursuit is defined as a response that is intentionally performed to achieve the desired state, and this behaviour is strongly related to internal motivation by expecting rewards that are contingent with achievement^1,7^. Here, when both the final goal and an immediate subgoal were present, the achievement of the sub-goal provided a milestone that indicated movement toward the goal and encouraged participants as compared to an LG-like situation, such as a long journey without milestones. Thus, the setting of subgoals should be used to enhance intrinsic motivation in goal maintenance and enhance self-efficacy^8^. This is the same mechanism as the final goal achievement enhancing goal achievement in the next task. If the FPC undergoes neuroplastic changes after goal achievement (as seen in L2L and LG-MoL), the SG-MoL setting should offer frequent opportunities for the FPC to undergo neuroplastic changes through multiple sets of miniature goal achievements. This may explain why SG-MoL induced the most robust neuroplastic changes.

In addition, the left FPC is involved in prospective memory, such as holding an intention toward future behaviour, checking future goals within the present situation, and dividing attention between planned actions and present activity^19,77^. In the case of action completion or occurrence of unforeseen events, these prospective plans may need to be updated or possibly reformulated. These processes may be among the core functions for future goal achievement. Detailed and systematic future examinations are needed in order to clarify the functional lateralization of the FPC in relation to goal-directed persistence.

This level of effect size has also been reported in previous studies^5^, including studies conducted by our group and several other laboratories^38,48,71,78^. The average age of participants in our study was 20 years old (early adulthood). Therefore, differences in the neuroplasticity of patients of different ages should be investigated in future studies.

Although multiple studies have confirmed the relationship between persistence and the FPC structure, our study is limited to the fact that we were unable to assert causality between these variables since we did not perform a knock-out experiment to prove that the disruption of FPC function increases Non-achievers. Additionally, we could not test if the pNA-Achiever converters can really achieve a goal on other occasions. To answer these questions, further research is required. To our knowledge, the present findings provide the first direct evidence of the high-level role and plasticity of the FPC in goal-directed persistence. The results also suggest that the neuroplastic changes in the left FPC are facilitated by appropriate coaching. We propose that the use of the FPC’s structure as an index of goal-directed persistence as well as coaching methods with subgoals may promote the development of new strategies that prevent people from straying from educational, social, and medical goals.

## Methods

### Subjects

We enrolled 65 subjects (36 men and 29 women) with a mean age of 22.5 years (SD = 5.3, range = 20–28) for the ToH experiment. In total, 81 subjects (40 males and 41 females) with a mean age of 23.0 years (SD = 3.5, range = 21–26) participated in the motor learning^79^ experiment for 1 month. We also reanalysed data from 47 participants (34 men and 33 women) with a mean age of 20.1 years (SD = 2.4, range = 19–22) from the training group (TG) and control group (CG) of a previous second language learning (L2L) experiment^1^. All subjects were selected through an interview and were highly motivated university students or graduates who were healthy and neurologically intact. None of the participants had a history of neuropsychiatric disorders, psychotropic medication use, or head injury. All participants provided written informed consent according to the study protocol, which was approved by the institutional review board (Advanced Telecommunication Research institute international and National Canter of Neurology and Psychiatry).

### Experimental design

Before the tasks or training, all participants who enrolled in the ToH, MOL, and L2L tasks underwent magnetic resonance imaging (MRI) scanning (T1-weighted images and diffusion-weighted images [DWI]) using a 3T MRI scanner (Siemens Trio, Erlangen, Germany). Participants who performed the MoL and L2L tasks also underwent MRI scanning after training and were assessed using the Wechsler Adult Intelligence Scale-3 (WAIS-3) and the NEO-Five Factor Inventory (NEO-FFI) in order to confirm basic intelligence and personality traits, respectively. They also reported their motivation using a 0–10 self-reported motivational scale. The personality description in the NEO-FFI is given in five dimensions: neuroticism, extraversion, agreeableness, openness, and conscientiousness. The Temperament and Character Inventory is a self-rated assessment that describes the following personality dimensions: novelty seeking, harm avoidance, reward dependence, persistence, self-directedness, cooperativeness, and self-transcendence. In the L2L experiment, we evaluated L2 abilities prior to training using the Test of English for International Communication. Participants in the MoL program, including those who dropped out, also underwent MRI scanning after the training period.

### Behavioral data acquisition

*Tower of Hanoi (ToH)*: participants completed the seven-disc version of the ToH on a computer program that was developed in-house using JAVA. Participants were presented with three pegs that held a stack of seven discs of graduated sizes and different colours on the screen. The goal was to move the stack of discs from the leftmost peg, one at a time, and replicate the stack on the rightmost peg. Placement of the discs was constrained by the rule that a larger ring could never be placed on top of a smaller one. The desired disc was moved by first clicking on it with a computer mouse to select it. Once selected, the disc could be moved to another peg using the mouse. A second click released the designated peg. If accomplished without any errors, the seven-disc puzzle can be completed in a minimum of 127 moves (two participants were aware of this). The participants were instructed to complete this puzzle in the fewest number of moves and as quickly as possible. There was no time restriction. However, if the participants expressed that they could not continue (a criterion that participants were unaware of), we ended the experiment and classified those participants as “Non-achievers”. Before starting the seven-disc ToH, participants practiced with a three-disc ToH in order to learn the rules. Participants were instructed to predict the time to complete the five- and seven-disc versions before the execution of the seven-disc ToH. We also interviewed all participants to confirm that they understood the rules and possessed a high motivation to complete the task according to a 0–10 self-reported motivational assessment scale and interview.

*Motor learning*: 81 participants underwent a 5-week computer-based training program that required them to learn and recall tapping sequences on a daily basis. The training program was developed in-house using JAVA. Eight keyboard buttons were assigned to eight locations (inline eight black circles) on a computer display. Each day, the participants were instructed to learn a new sequence of 10 tapping movements (trial-and-error phase) and combine it with the sequences recollected from all previous days. When participants hit the correct key during the trial-and-error phase, the black circle became red; when they missed the key, it turned blue. Thus, the combined sequence grew daily to a total length of 250 tapping movements by the end of the training period. Each day’s training was completed if participants could memorize the daily quota (all previous sequences plus new sequences) and tap it to a 2-Hz metronome with no mistakes (test-phase). Participants were allowed to keep trying until they could perform the daily quota perfectly.

Before training, participants were classified as Achievers or Non-achievers by the persistency detector (see below, persistency detector) and placed into two groups that were provided with a goal at either a short interval (SG) (*n* = 41) or a long interval (LG) (*n* = 40). There were no significant differences in the ratio of predicted Achievers/Non-achievers, sex, or age. Participants in the SG were presented with the word “clear” on the computer screen when they gained two or three sequential tapping successes, both in the trial-and-error phase and test phase. Participants in the LG group were presented with “clear” only after gaining 10 tapping successes (one clear showing in one day), both in the trial-and-error phase and test phase. There were no pauses in showing the word “clear”; thus, the learning content was the same across the groups. Participants who announced their resignation or neglected training for at least three consecutive days were classified as Non-achievers. Participants were allowed to perform two daily programs in one day. None of the participants restarted training after neglecting it for several days. Self-reported reasons for dropping out are shown in Supplementary Table 2.

### Time prediction

In both the ToH and MoL experiments, participants were instructed to predict the time required to complete the tasks. In the ToH experiment, participants were asked to predict the time it would take to complete five to seven discs before the execution of the seven-disc ToH. All participants were able to complete five discs before the seven-disc ToH, whereas only half could complete seven discs. Therefore, we used the predicted and actual time of the five-disc ToH to measure the differences in the prediction of performance. In the MoL, participants were instructed to predict the completion time of each daily program, which allowed us to measure the differences in the prediction of daily performance during the learning period.

### Image data acquisition

MRI data were acquired using a 3-Tesla MRI scanner with an eight-channel phased-array receiver coil (Siemens Trio, Erlangen, Germany). High-resolution, three-dimensional (3D), T1-weighted anatomical images were obtained with a magnetization-prepared rapid gradient echo sequence designed as follows: repetition time (TR) = 2000 ms, echo time (TE) = 4.4 ms, inversion time (TI) = 990 ms, flip angle = 80°, matrix size = 192 × 176, field of view (FOV) = 192 × 176 mm, and 1 mm^3^ isotropic voxels. We also acquired whole-brain DWI as follows: TR = 7900 ms, TE = 80 ms, 65 slices, flip angle = 90°, matrix size = 96 × 96, FOV = 192 × 192 mm, 2 × 2 × 2 mm^3^ isotropic voxels, 81 volumes with diffusion weighting (*b* value = 700 s mm^−2^) for different motion probing gradient directions and nine volumes without diffusion weighting (*b* = 0 s mm^−2^). Field-map images were acquired in the same scanning space as that of the DWI (TE1 = 5.19 ms; TE2 = 7.65 ms). All image data were converted into the Neuroimaging Informatics Technology Initiative format before further processing.

## Statistics and reproducibility

### Image data analysis

*GM-VBM*: high-resolution 3D: T1-weighted images were subjected to voxel-based morphometry (VBM) analysis using the VBM8 toolbox (http://dbm.neuro.uni-jena.de/vbm.html) implemented in SPM8 (http://www.fil.ion.ucl.ac.uk/spm). The pre-processing steps were as follows^5^: (1) each image was segmented into gray matter (GM), white matter (WM), and cerebrospinal fluid images in the native image space; (2) diffeomorphic anatomical registration using the exponentiated Lie algebra (DARTEL) registration method was used to create a study-specific mean GM image template using the aligned images from all the participants to improve inter-subject registration; (3) individual GM images were registered to the study-specific mean GM template; (4) the registered GM images were further transformed into the Montreal Neurological Institute (MNI) space; and (5) these normalized GM images were smoothed using a Gaussian kernel of 12 mm full-width at half-maximum.

For the ToH experiment, we tested the hypothesis that particular brain regions were reserved for completing the task independent of task content or duration. If this hypothesis was correct, participants with particularly developed structures in the brain would show goal-directed persistence. To test this hypothesis, we performed a two-sample *t*-test using MRI data from the pre-task condition in Achievers and Non-achievers. We performed a general linear model (GLM) analysis for the ToH and MoL tasks that incorporated sex, age, motivation score, IQ, and prediction time as covariates to remove their confounding effects (*p* < 0.05, FWE-corrected). For L2L, we performed a GLM analysis for the L2L task that incorporated only sex, age, and IQ as covariates. To identify changes in GM induced by MoL training, we conducted a two-by-two mixed repeated-measures analysis of variance using the time (Pre and Post) as a within-subject variable and group (Achievers and Non-achievers) as a between-subjects variable.

*DWI-TBSS*: data pre-processing and analysis of DWI were performed using the Oxford Centre for Functional MRI of the Brain (FMRIB) software library (FSL 4.1; http://www.fmrib.ox.ac.uk/fsl/). All DWI were registered to the b0 images. Nonlinear image distortions due to the magnetic field (b0) inhomogeneity were corrected based on the field-map images using FUGUE in the FMRIB software library. The registered images were skull-stripped using the Brain Extraction Tool. FA maps were calculated using FMRIB Diffusion Toolbox v2.0. After calculating the FA map for each participant, we implemented a voxel-wise statistical analysis of the FA data using TBSS v1.2. In brief, TBSS was performed as follows: (1) alignment of the FA images from all participants to a template that was arbitrarily selected from those FA images, (2) transformation of all aligned FA images into MNI space using affine registration to remove the effect of cross-subject spatial variability, (3) creation of a mean FA image and FA skeleton images corresponding to the centre of the WM using a threshold of FA _0.20, (4) projection of the individual FAs onto the mean FA skeleton, and (5) voxel-wise cross-subject statistical analyses.

Similar to the VBM analysis, we first tested whether the WM structure before each task could predict whether participants would complete the task. To identify the learning-induced reorganization of WM during the MoL program, we conducted a two-by-two mixed repeated-measures ANOVA using time as a within-subject variable and group as a between-subject variable (*p* < 0.05, FWE-corrected), yielding FA changes specific to the MoL training program.

### Persistency detector

We applied classification analysis to confirm the domain-generality of the FPC region associated with goal achievement. We extracted regional features from the brain regions that exhibited significant group differences between the Achievers and the Non-achievers in both GM (*x* = −22, *y* = 57, *z* = 22) and WM (*x* = −19, *y* = 50, *z* = 10) using a 5 mm spherical volume of interest (VOI) at peak voxels for ToH. A Fisher’s linear classifier was trained based on these data and classified the Achievers and the Non-achievers based on whether its output value was positive or negative, respectively. Then, we applied leave-one-out cross-validation within the ToH data to discriminate Achievers from Non-achievers from pre- and post-L2L data and pre- and post-MOL data. We calculated the precision, recall, true positive rate, and false positive rate for LG in ToH and SG in MoL and L2L (Table 4). Moreover, to strengthen the accuracy of the persistency detector by removing the nature of tasks and reducing over fitting, the Fisher’s Linear Discriminant was performed as follows: 61 samples were randomly allocated into groups of 43 (70%) and 18 (30%). The first group (70% of the samples) was designated as training data and the second group (30% of the samples) was designated as test data. Evaluation of the discriminant method was performed using the bootstrap method (1000 times).

### VOI analysis

To investigate how SG and LG differentially induced neuroplastic changes in the FPC structure, we retrieved VOIs before and after MoL with a 5 mm radius over the medial prefrontal cortex (mPFC GM: x, *y*, z = 28, 58, 18, respectively; FA: *x*, y, *z* = 25, 48, 8, respectively), which has been shown to be involved in a category-independent goal value signal^5^ and yields the learning-induced change rate of GM and FA (post-pre/pre). We performed a two-way ANOVA with STRATEGY (LG and SG) and PERSISTENCE (Achiever and Non-Achiever after learning) as between-subject factors. After obtaining participant consent, we obtained structural MRI data from all participants using pre- and post-MoL imaging sessions. In addition, we performed two-sample t-tests using the same mPFC VOIs on the Achievers in the L2L program and controls that did not complete the L2L program to test the generalizability of learning-induced neuroplasticity in the FPC. A two-way ANOVA using PREDICTION (pA and pNA) and STRATEGY (LG and SG) as factors was performed for mPFC VOIs of GM and FA.

The analysis outline throughout the experiment was shown in Supplementary Fig. 2.

### Reporting summary

Further information on research design is available in the Nature Research Reporting Summary linked to this article.

## Supplementary information

Supplementary Information

Supplementary Data 1

Reporting Summary

Description of Additional Supplementary Files

## Data Availability

The data that supports the findings of this study is available upon request to the corresponding author (for verification purposes only and not for future studies). Concerning the raw brain imaging data, two participants did not agree to share, and thus data from these two participants is not available.

## Code availability

The code that supports the findings of this study is available upon request to the corresponding author (for verification purposes only).

## References

[1] Wiese, B. S. *Successful pursuit of personal goals and subjective well-being*. (Erlbaum., Hillsdale, 2007) .

[2] Curioni CC, Lourenço PM (2005). Long-term weight loss after diet and exercise: a systematic review. Int J. Obes. (Lond.).

[3] Zhou X (2009). Attempts to quit smoking and relapse: factors associated with success or failure from the ATTEMPT cohort study. Addict. Behav..

[4] Carmody TP, Senner JW, Malinow MR, Matarazzo JD (1980). Physical exercise rehabilitation: long-term dropout rate in cardiac patients. J. Behav. Med..

[5] Hosoda C, Tanaka K, Nariai T, Honda M, Hanakawa T (2013). Dynamic neural network reorganization associated with second language vocabulary acquisition: a multimodal imaging study. J. Neurosci..

[6] Ericsson KA (2008). Deliberate practice and acquisition of expert performance: a general overview. Acad. Emerg. Med..

[7] Gusnard DA (2003). Persistence and brain circuitry. Proc. Natl Acad. Sci. USA.

[8] Baumeister RF (2003). Ego depletion and self-regulation failure: a resource model of self-control. Alcohol Clin. Exp. Res..

[9] Beauregard, M., Levesque, J. & Paquette, V. in *Consciousness, Emotional Self-Regulation and the Brain* (ed Mario Beauregard). (John Benjamins B.V., 2003).

[10] Barber L, Grawitch MJ, Munz DC (2013). Are better sleepers more engaged workers? A self-regulatory approach to sleep hygiene and work engagement. Stress Health.

[11] Baumeister RF, Gailliot M, DeWall CN, Oaten M (2006). Self-regulation and personality: how interventions increase regulatory success, and how depletion moderates the effects of traits on behaviour. J. Pers..

[12] Muraven M, Baumeister RF (2000). Self-regulation and depletion of limited resources: does self-control resemble a muscle?. Psychol. Bull..

[13] Muraven M, Baumeister RF, Tice DM (1999). Longitudinal improvement of self-regulation through practice: building self-control strength through repeated exercise. J. Soc. Psychol..

[14] Shalley C, Oldham GR (1985). Effects of goal difficulty and expected external evaluation on intrinsic motivation: a laboratory study. Acad. Manag. J..

[15] Duckworth AL, Peterson C, Mattews MD, Kelly DR (2007). Grit: Perseverance and passion for long-term goals. J. Pers. Soc. Psychol..

[16] Martin, L. L. & Tesser, A. *Striving and Feeling: Interactions Among Goals, Affect, and Self-regulation*. (Psychology Press, 1996).

[17] Fishbach A, Dhar R, Zhang Y (2006). Subgoals as substitutes or complements: The role of goal accessibility. J. Pers. Soc. Psychol..

[18] Stock J, Cervone D (1990). Proximal goal-setting and self-regulatory processes. Cogn. Ther. Res.

[19] Burgess PW, Scott SK, Frith CD (2003). The role of the rostral frontal cortex (area 10) in prospective memory: a lateral versus medial dissociation. Neuropsychologia.

[20] Homskaya ED (1973). The human frontal lobes and their role in the organization of activity. Acta Neurobiol. Exp. (Wars.).

[21] Burgess PW (2000). Strategy application disorder: the role of the frontal lobes in human multitasking. Psychol. Res..

[22] Collins A, Koechlin E (2012). Reasoning, learning, and creativity: frontal lobe function and human decision-making. PLoS Biol..

[23] Hoffmann M (2013). The human frontal lobes and frontal network systems: an evolutionary, clinical, and treatment perspective. ISRN Neurol..

[24] Montgomery SH (2013). The human frontal lobes: not relatively large but still disproportionately important? A commentary on Barton and Venditti. Brain Behav. Evol..

[25] Badre D, Nee DE (2018). Frontal cortex and the hierarchical control of behaviour. Trends Cogn. Sci..

[26] Okuda J (2007). Differential involvement of regions of rostral prefrontal cortex (Brodmann area 10) in time- and event-based prospective memory. Int J. Psychophysiol..

[27] Boorman ED, Behrens TE, Woolrich MW, Rushworth MF (2009). How green is the grass on the other side? Frontopolar cortex and the evidence in favor of alternative courses of action. Neuron.

[28] Burke CJ, Brünger C, Kahnt T, Park SQ, Tobler PN (2013). Neural integration of risk and effort costs by the frontal pole: only upon request. J. Neurosci..

[29] Daw ND, O’Doherty JP, Dayan P, Seymour B, Dolan RJ (2006). Cortical substrates for exploratory decisions in humans. Nature.

[30] Sakai K, Passingham RE (2003). Prefrontal interactions reflect future task operations. Nat. Neurosci..

[31] Qiu L (2018). The neural system of metacognition accompanying decision-making in the prefrontal cortex. PLoS Biol..

[32] Tsujimoto S, Genovesio A, Wise SP (2011). Frontal pole cortex: encoding ends at the end of the endbrain. Trends Cogn. Sci..

[33] Koechlin E, Basso G, Pietrini P, Panzer S, Grafman J (1999). The role of the anterior prefrontal cortex in human cognition. Nature.

[34] Desrochers TM, Chatham CH, Badre D (2015). The necessity of rostrolateral prefrontal cortex for higher-level sequential behaviour. Neuron.

[35] Mahncke HW (2006). Memory enhancement in healthy older adults using a brain plasticity-based training program: a randomized, controlled study. Proc. Natl Acad. Sci. USA.

[36] Boltzmann M, Mohammadi B, Samii A, Munte TF, Rüsseler J (2017). Structural changes in functionally illiterate adults after intensive training. Neuroscience.

[37] Boyke J, Driemeyer J, Gaser C, Büchel C, May A (2008). Training-induced brain structure changes in the elderly. J. Neurosci..

[38] Scholz J, Klein MC, Behrens TE, Johansen-Berg H (2009). Training induces changes in white-matter architecture. Nat. Neurosci..

[39] Habibi A (2018). Childhood music training induces change in micro and macroscopic brain structure: Results from a longitudinal study. Cereb. Cortex.

[40] Sagi Y (2012). Learning in the fast lane: new insights into neuroplasticity. Neuron.

[41] Yotsumoto Y (2014). White matter in the older brain is more plastic than in the younger brain. Nat. Commun..

[42] Brandon TH (2003). Pretreatment task persistence predicts smoking cessation outcome. J. Abnorm Psychol..

[43] Archambault I, Janosz M, Fallu JS, Pagani LS (2009). Student engagement and its relationship with early high school dropout. J. Adolesc..

[44] Goel V, Grafman J (1995). Are the frontal lobes implicated in “planning” functions? Interpreting data from the Tower of Hanoi. Neuropsychologia.

[45] Treadway MT (2012). Dopaminergic mechanisms of individual differences in human effort-based decision-making. J. Neurosci..

[46] Burgess PW, Veitch E, de Lacy Costello A, Shallice T (2000). The cognitive and neuroanatomical correlates of multitasking. Neuropsychologia.

[47] Burgess PW, Gilbert SJ, Dumontheil I (2007). Function and localization within rostral prefrontal cortex (area 10). Philos. Trans. R. Soc. Lond. B Biol. Sci..

[48] Draganski B (2004). Neuroplasticity: changes in grey matter induced by training. Nature.

[49] Driemeyer J, Boyke J, Gaser C, Büchel C, May A (2008). Changes in gray matter induced by learning-revisited. PLoS One.

[50] Mansouri FA, Koechlin E, Rosa MGP, Buckley MJ (2017). Managing competing goals—a key role for the frontopolar cortex. Nat. Rev. Neurosci..

[51] Soutschek A, Kang P, Ruff CC, Hare TA, Tobler PN (2018). Brain stimulation over the frontopolar cortex enhances motivation to exert effort for reward. Biol. Psychiatry.

[52] Ariely D, Wertenbroch K (2002). Procrastination, deadlines, and performance: self-control by precommitment. Psychol. Sci..

[53] Crockett MJ (2013). Restricting temptations: neural mechanisms of precommitment. Neuron.

[54] McClure SM, Laibson DI, Loewenstein G, Cohen JD (2004). Separate neural systems value immediate and delayed monetary rewards. Science.

[55] Metcalfe J, Mischel W (1999). A hot/cool-system analysis of delay of gratification: dynamics of willpower. Psychol. Rev..

[56] Shallice T, Burgess PW (1991). Deficits in strategy application following frontal lobe damage in man. Brain.

[57] Bechara A, Tranel D, Damasio H, Damasio AR (1996). Failure to respond autonomically to anticipated future outcomes following damage to prefrontal cortex. Cereb. Cortex.

[58] Donald, M. *Origins of the Modern Mind: Three Stages in the Evolution of Culture and Cognition*. (Harvard University Press, 1991).

[59] Ericsson KA (2015). Acquisition and maintenance of medical expertise: a perspective from the expert-performance approach with deliberate practice. Acad. Med..

[60] Corrigendum: Deliberate practice and performance in music, games, sports, education, and professions: a meta-a nalysis. *Psychol. Sci.* **29**, 1202-1204, 10.1177/0956797618769891 (2018). 10.1177/0956797618769891 29733772

[61] Macnamara BN, Hambrick DZ, Oswald FL (2014). Deliberate practice and performance in music, games, sports, education, and professions: a meta-analysis. Psychol. Sci..

[62] Badre D, D’Esposito M (2009). Is the rostro-caudal axis of the frontal lobe hierarchical?. Nat. Rev. Neurosci..

[63] Tsujimoto S, Genovesio A (2017). Firing variability of frontal pole neurons during a cued strategy task. J. Cogn. Neurosci..

[64] Tsujimoto S, Genovesio A, Wise SP (2010). Evaluating self-generated decisions in frontal pole cortex of monkeys. Nat. Neurosci..

[65] Koechlin E (2011). Frontal pole function: what is specifically human?. Trends Cogn. Sci..

[66] Azuar C (2014). Testing the model of caudo-rostral organization of cognitive control in the human with frontal lesions. Neuroimage.

[67] Pochon JB (2002). The neural system that bridges reward and cognition in humans: an fMRI study. Proc. Natl Acad. Sci. USA.

[68] Elliot AJ (1999). Approach and avoidance motivation and achievement goals. Educ. Psychol.-Us.

[69] Steele CJ, Scholz J, Douaud G, Johansen-Berg H, Penhune VB (2012). Structural correlates of skilled performance on a motor sequence task. Front Hum. Neurosci..

[70] Tuch DS (2005). Choice reaction time performance correlates with diffusion anisotropy in white matter pathways supporting visuospatial attention. Proc. Natl Acad. Sci. USA.

[71] Taubert M (2010). Dynamic properties of human brain structure: learning-related changes in cortical areas and associated fibre connections. J. Neurosci..

[72] Elliot AJ (2006). The hierarchical model of approach-avoidance motivation. Motiv Emot..

[73] Carver CS (2018). Control theory, goal attainment, and psychopathology. Psychol. Inq..

[74] Miller GA, Crocker LD, Spielberg JM, Infantolino ZP, Heller W (2013). Issues in localization of brain function: the case of lateralized frontal cortex in cognition, emotion, and psychopathology. Front Integr. Neurosci..

[75] Wimmer S, Lackner HK, Papousek I, Paechter M (2018). Goal orientations and activation of approach versus avoidance motivation while awaiting an achievement situation in the laboratory. Front Psychol..

[76] Burgess PW, Gonen-Yaacovi G, Volle E (2011). Functional neuroimaging studies of prospective memory: what have we learnt so far?. Neuropsychologia.

[77] Landi SM, Baguear F, Della-Maggiore V (2011). One week of motor adaptation induces structural changes in primary motor cortex that predict long-term memory one year later. J. Neurosci..

[78] Oermann MH, Molloy MA, Vaughn J (2015). Use of deliberate practice in teaching in nursing. Nurse Educ. Today.

[79] Wood AM (2018). Risk thresholds for alcohol consumption: combined analysis of individual-participant data for 599 912 current drinkers in 83 prospective studies. Lancet.

