# Supplementary material for: Plastic frontal pole cortex structure related to individual persistence for goal achievement
Source: Commun Biol. 2020 Apr 28;3:194. doi: 10.1038/s42003-020-0930-4 (PMC7189238; doi:10.1038/s42003-020-0930-4)
Supplement: Supplementary file 1 — Supplementary Information [file 42003_2020_930_MOESM1_ESM.pdf]

Supporting Information

Supplementary Figure 1. Differences between Achievers and Non-achievers in each experiment.

The difference between the Achievers and the Non-achievers in MoL (a. FA b.GM) and in MoL (c.FA d.GM)

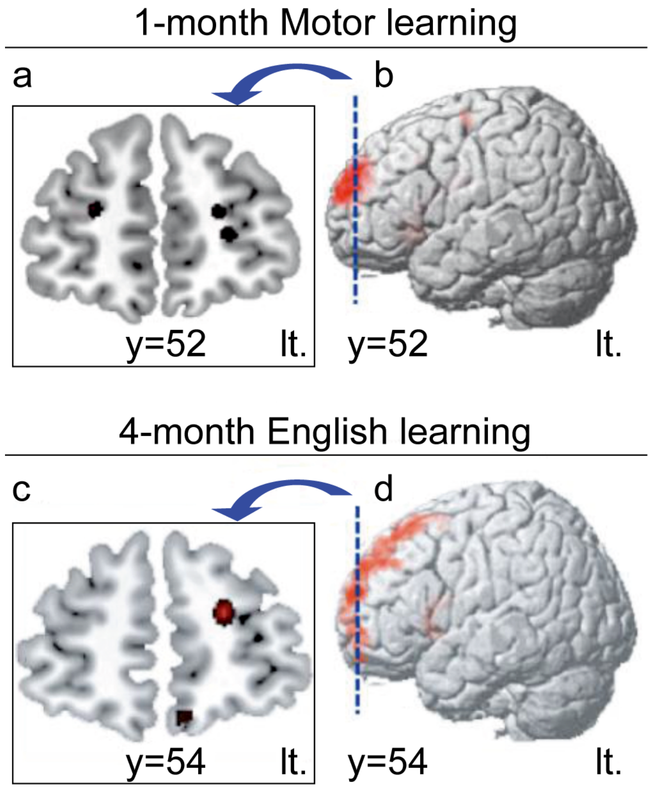

The coordinates (**x**, **y**, **z**) indicate local maxima in each brain region according to the MNI template.

Supplementary Figure 2.

The figure to the analysis outline throughout the experiment.

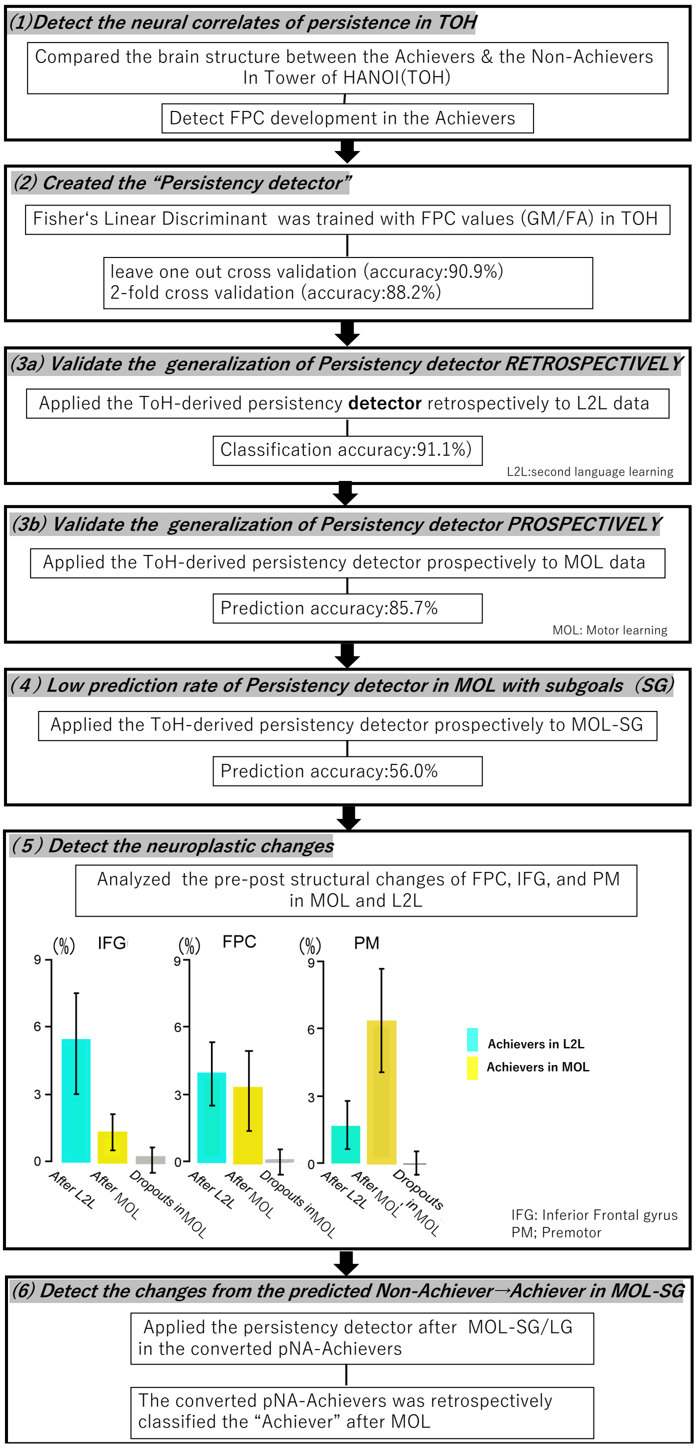

Supplementary Table 1.

Differences between the Achievers and the Non-achievers for each experiment

Anatomical location	Coordinates			Z-value	P corrected
	x	y	z		
TOH experiment					
Left superior frontal gyrus	-22	57	22	6.34	0.01 0.020
L2L experiment					
Left superior frontal gyrus	-12	58	34	4.66	0.01
MoL experiment					
Left superior frontal gyrus	-16	50	22	5.58	0.01

The coordinates (**x**, **y**, **z**) indicate local maxima in each brain region according to the MNI template.

Supplementary Table2.

Classification accuracy of the persistency detector by leave-one-out cross-validation.

	L2L	TOH	VML
Tower of Hanoi-based classifier by grey matter volume and FA value	80.7%	90.9%	80.0%
Tower of Hanoi-based classifier by grey matter volume	55.6%	62.4%	54.3%
Tower of Hanoi-based classifier by FA value	51.1%	58.2%	52.7%

The results show a relationship between the FPC region and goal-achievement for various behavioural domains, including executive control, motor learning, and language learning, as well as for different durations of engagement (from tens of minutes to several weeks).

Supplementary Table 3.

Precision and recall rate of persistency detector:

	TOH	LG in MoL	SG in MoL	L2L
Precision	91.2%	84.2%	100.0%	88.0%
Recall	91.2%	84.2%	52.6%	95.7%
True Positive rate	91.2%	84.2%	52.6%	95.7%
False Positive rate	9.7%	13.6%	0%	13.4%

Supplementary Table 4.

Reasons for not completing the tasks

Number of subjects	Reason for dropout
Tower of Hanoi	
28	Harder than expected
3	Bored and tired
Motor Learning	
19	Harder than expected
2	Became busy
2	Bored
1	Eyes hurting

Supplementary Table 5.

Precision and Recall rate of persistent detector using TOH, L2L, and MOT(LG) data as one dataset

Precision	True positive rate	True Negative Rate	False positive Rate	False positive Rate
90.6%	88.9%	90.4%	11.1%	9.6%
