# Supplementary material for: Plastic frontal pole cortex structure related to individual persistence for goal achievement
Source: Commun Biol. 2020 Apr 28;3:194. doi: 10.1038/s42003-020-0930-4 (PMC7189238; doi:10.1038/s42003-020-0930-4)
Supplement: Supplementary file 3 — Reporting Summary [file 42003_2020_930_MOESM3_ESM.pdf]

nature research

Corresponding author(s): Chihiro Hosoda

Last updated by author(s): 2020.1.17

Reporting Summary

Nature Research wishes to improve the reproducibility of the work that we publish. This form provides structure for consistency and transparency in reporting. For further information on Nature Research policies, see Authors & Referees and the Editorial Policy Checklist.

Statistics

For all statistical analyses, confirm that the following items are present in the figure legend, table legend, main text, or Methods section.

n/a	Confirmed	
☐	☒	The exact sample size (n) for each experimental group/condition, given as a discrete number and unit of measurement
☐	☒	A statement on whether measurements were taken from distinct samples or whether the same sample was measured repeatedly
☐	☒	The statistical test(s) used AND whether they are one- or two-sided<br>*Only common tests should be described solely by name; describe more complex techniques in the Methods section.*
☐	☒	A description of all covariates tested
☐	☒	A description of any assumptions or corrections, such as tests of normality and adjustment for multiple comparisons
☐	☒	A full description of the statistical parameters including central tendency (e.g. means) or other basic estimates (e.g. regression coefficient) AND variation (e.g. standard deviation) or associated estimates of uncertainty (e.g. confidence intervals)
☐	☒	For null hypothesis testing, the test statistic (e.g. F, t, r) with confidence intervals, effect sizes, degrees of freedom and P value noted<br>*Give P values as exact values whenever suitable.*
☒	☐	For Bayesian analysis, information on the choice of priors and Markov chain Monte Carlo settings
☒	☐	For hierarchical and complex designs, identification of the appropriate level for tests and full reporting of outcomes
☐	☒	Estimates of effect sizes (e.g. Cohen's d, Pearson's r), indicating how they were calculated

Our web collection on statistics for biologists contains articles on many of the points above.

Software and code

Policy information about availability of computer code

Data collection	No software was used
Data analysis	High-resolution 3D T1-weighted images were subjected to voxel-based morphometry (VBM) analysis using the VBM8 toolbox implemented in SPM8. DWI were performed using the Oxford Centre for Functional MRI of the Brain (FMRIB) software library (FSL 4.1)

For manuscripts utilizing custom algorithms or software that are central to the research but not yet described in published literature, software must be made available to editors/reviewers. We strongly encourage code deposition in a community repository (e.g. GitHub). See the Nature Research guidelines for submitting code & software for further information.

Data

Policy information about availability of data

All manuscripts must include a data availability statement. This statement should provide the following information, where applicable:

- Accession codes, unique identifiers, or web links for publicly available datasets
- A list of figures that have associated raw data
- A description of any restrictions on data availability

The datasets generated during and/or analysed during the current study are available from the corresponding author on reasonable request.

Field-specific reporting

Please select the one below that is the best fit for your research. If you are not sure, read the appropriate sections before making your selection.

☐ Life sciences ☒ Behavioural & social sciences ☐ Ecological, evolutionary & environmental sciences

Behavioural & social sciences study design

All studies must disclose on these points even when the disclosure is negative.

Study description	Quantitative cross sectional study (Tower of HANOI) and the longitudinal studies (Motor learning).
Research sample	We enrolled 65 subjects (36 males and 29 females) with a mean age of 22.5 years (SD = 5.3, range = 20–28) for the Tower of Hanoi (ToH) experiment. In total, 81 subjects (40 males and 41 females) with a mean age of 23.0 years (SD = 3.5, range = 21–26) participated in the motor learning 37 experiment for 1 month. Subjects were all Japanese university students.
Sampling strategy	We collected the subjects randomly and no sample size calculation was performed.
Data collection	All participants underwent magnetic resonance imaging (MRI) scanning (T1-weighted images and diffusion-weighted images [DWI]) using a 3T MRI scanner (Siemens Trio, Erlangen, Germany) by the researcher. Also they underwent the using the Wechsler Adult Intelligence Scale-3 (WAIS-3) and the NEO-Five Factor Inventory (NEO-FFI) by the well-trained clinical psychologist.
Timing	The experiment of Tower of HANOI was started January, 2012 and ended in March. The The experiment of Motor learning was started January, 2013 and ended in July.
Data exclusions	No data was excluded because we analyzed the data of dropouts.
Non-participation	31 were dropped out from the Experiment of Tower of Hanoi and 27 were dropped out from the Experiment of Motor learning
Randomization	The subjects no allocated the groups

Reporting for specific materials, systems and methods

We require information from authors about some types of materials, experimental systems and methods used in many studies. Here, indicate whether each material, system or method listed is relevant to your study. If you are not sure if a list item applies to your research, read the appropriate section before selecting a response.

Materials & experimental systems

n/a	Involved in the study	
☒	☐	Antibodies
☒	☐	Eukaryotic cell lines
☒	☐	Palaeontology
☒	☐	Animals and other organisms
☐	☒	Human research participants
☒	☐	Clinical data

Methods

n/a	Involved in the study	
☒	☐	ChIP-seq
☒	☐	Flow cytometry
☐	☒	MRI-based neuroimaging

Human research participants

Policy information about studies involving human research participants

Population characteristics	We enrolled 65 subjects (36 males and 29 females) with a mean age of 22.5 years (SD = 5.3, range = 20–28) for the Tower of Hanoi (ToH) experiment. In total, 81 subjects (40 males and 41 females) with a mean age of 23.0 years (SD = 3.5, range = 21–26) participated in the motor learning 37 experiment for 1 month. All were healthy and neurologically intact, with no history of neuropsychiatric disorders,psychotropic medication use, or head injury.
Recruitment	Recruitment was randomly invited using an advertisement describing the details of the experiments posted on the university bulletin board.
Ethics oversight	ATR Computational Neuroscience Laboratories and National Center of Neurology and Psychiatry,

Note that full information on the approval of the study protocol must also be provided in the manuscript.

Magnetic resonance imaging

Experimental design

Design type	Structural MRI (T1-weighted anatomical images and Diffusion-weighted imaging)

Design specifications | No tasks while the structural MRI was obtained

Behavioral performance measures | No tasks while the structural MRI was obtained. Out side of th MRI, we measured whether the subjects complete the assigned tasks or not.

Acquisition

Imaging type(s) | T1-weighted anatomical images and Diffusion-weighted imaging

Field strength | 3-T

Sequence & imaging parameters | Repetition time (TR) = 2000 ms, echo time (TE) = 4.4 ms, inversion time (TI) = 990 ms, flip angle = 80°, matrix size = 192 × 176, field of view (FOV) = 192 × 176 mm, and 1 mm3 isotropic voxels

Area of acquisition | A whole brain scan was used

Diffusion MRI ☒ Used ☐ Not used

Parameters | TR = 7900 ms, TE = 80 ms, 65 slices, flip angle = 90°, matrix size = 96 × 96, FOV = 192 × 192 mm, 2 × 2 × 2 mm3 isotropic voxels, 81 volumes with diffusion weighting (b value = 700 s/mm2) for different motion probing gradient directions, and nine volumes without diffusion weighting (b = 0 s/mm2)

Preprocessing

Preprocessing software | High-resolution 3D T1-weighted images were subjected to voxel-based morphometry (VBM) analysis using the VBM8 toolbox (http://dbm.neuro.uni-jena.de/vbm.html) implemented in SPM8 (http://www.fil.ion.ucl.ac.uk/spm).

Normalization | In the SPM8 plus DARTEL procedure, T1-weighted images were classified into gray matter, white matter and CSF using the segmentation routine implemented in SPM8, that gives both the native space versions and DARTEL imported versions of the tissues. DARTEL works by aligning gray matter among the images, while simultaneously aligning white matter. This is achieved by generating increasingly crisp average template data, to which the data are iteratively aligned. The flow fields and final template image created in the previous step are used to generate smoothed (12-mm FWHM), modulated, spatially normalized and Jacobian scaled gray and white matter images resliced to isotropic voxel size in Montreal Neurological Institute space.

Normalization template | The Montreal Neurological Institute (MNI) space

Noise and artifact removal | VBM8 apply two denoising methods. The first method is a spatially adaptive nonlocal means (SANLM) denoising filter (Manjon et al. 2010). This filter will remove noise while preserving edges and is implemented as preprocessing step. The second method is a classical Markov Random Field (MRF) approach, which incorporates spatial prior information of adjacent voxels into the segmentation estimation

Volume censoring | *Define your software and/or method and criteria for volume censoring, and state the extent of such censoring.*

Statistical modeling & inference

Model type and settings | For the Tower of HANOI and Motor learning, we performed a two-sample t-test incorporating sex, age, motivation score, IQ, and prediction time as covariates to remove their confounding effects using MRI data from the pre-task condition in Achievers and Non-achievers. To identify changes induced by motor training, we conducted a two-by-two mixed repeated-measures analysis of variance with time (Pre and Post) as a within-subject variable and group (Achievers and Non-achievers) as a between-subjects variable.

Effect(s) tested | *Define precise effect in terms of the task or stimulus conditions instead of psychological concepts and indicate whether ANOVA or factorial designs were used.*

Specify type of analysis: ☒ Whole brain ☐ ROI-based ☐ Both

Statistic type for inference (See Eklund et al. 2016) | Voxel wise were used

Correction | FWE correction were used

Models & analysis

n/a	Involved in the study
☒	☐ Functional and/or effective connectivity
☒	☐ Graph analysis
☐	☒ Multivariate modeling or predictive analysis

Multivariate modeling and predictive analysis | We applied classification analysis to confirm the domain-generality of the FPC region associated with goal achievement. Using a 5-mm spherical volume of interest (VOI) at peak voxels for ToH, we extracted

regional features from the brain regions exhibiting significant group differences between the Achievers and the Non-achievers in both GM (x = –22, y = 57, z = 22) and WM (x = –19, y = 50, z = 10). A linear classifier was trained based on these data and classified the Achievers and the Non-achievers based on whether its output value was positive or negative, respectively. We then applied leave-one-out cross-validation within the Tower of HANOI data to discriminate Achievers from Non-achievers from pre- and post-motor learning data.
