# Supplementary material for: Plastic frontal pole cortex structure related to individual persistence for goal achievement
Source: Commun Biol. 2020 Apr 28;3:194. doi: 10.1038/s42003-020-0930-4 (PMC7189238; doi:10.1038/s42003-020-0930-4)
Supplement: Supplementary file 4 — Description of Additional Supplementary Files [file 42003_2020_930_MOESM4_ESM.pdf]

Description of Additional Supplementary Files

File Name: **Supplementary Data 1**
Description: Data of mean and standard deviation of subject demographics, IQ, personality assessments, and self-reported motivation
